# Insulin Receptor Isoform Variations in Prostate Cancer Cells

**DOI:** 10.3389/fendo.2016.00132

**Published:** 2016-09-28

**Authors:** Claire M. Perks, H. A. Zielinska, Jing Wang, Caroline Jarrett, A. Frankow, Michael R. Ladomery, Amit Bahl, Anthony Rhodes, Jon Oxley, Jeff M. P. Holly

**Affiliations:** ^1^IGFs and Metabolic Endocrinology Group, School of Clinical Sciences, Southmead Hospital, University of Bristol, Bristol, UK; ^2^Department of Biological, Biomedical and Analytical Sciences, Faculty of Health and Applied Sciences, University of the West of England, Bristol, UK; ^3^Department of Clinical Oncology, Bristol Haematology and Oncology Centre, University Hospitals Bristol, Bristol, UK; ^4^Department of Pathology, Faculty of Medicine, University of Malaya, Kuala Lumpur, Malaysia; ^5^Department of Cellular Histopathology, North Bristol NHS Trust, Bristol, UK

**Keywords:** prostate cancer, insulin receptor, IGF-II, insulin, splicing variants

## Abstract

Men who develop prostate cancer (PCa) increasingly have one of the co-morbidities associated with a Western lifestyle that are characterized by hyperinsulinemia, hyperglycemia and increased expression of insulin-like growth factors-I (IGF-I) and IGF-II. Each have been associated with poor prognosis and more aggressive cancers that exhibit increased metabolism and increased glucose uptake. The insulin receptor (IR) has two splice isoforms IR-A and IR-B: IR-A has a higher affinity for IGF-II comparable to that for insulin, whereas the IR-B isoform predominantly just binds to insulin. In this study, we assessed alterations in the IR-A and IR-B isoform ratio and associated changes in cell proliferation and migration of PCa cell lines following exposure to altered concentrations of glucose and treatment with IGF-II and insulin. We observed that where IR-B predominated insulin had a greater effect on migration than IGF-II and IGF-II was more effective when IR-A was the main isoform. With regard to proliferation IGF-II was more effective than insulin regardless of which isoform was dominant. We assessed the abundance of the IR isoforms both *in vivo* and *in vitro* and observed that the majority of the tissue samples and cell lines expressed more IR-A than IR-B. Alterations in the isoforms in response to changes in their hormonal milieu could have a profound impact on how malignant cells behave and play a role in promoting carcinogenesis. A greater understanding of the mechanisms underlying changes in alternative splicing of the IR may provide additional targets for future cancer therapies.

## Introduction

Following cloning of the insulin receptor (IR) cDNA by both Ullrich et al. and Ebina et al. in 1985, it was observed that the cDNAs were of different lengths suggesting that the IR existed in two different forms (1, 2). Later defined as isoform A (IR-A), in which exon 11 is skipped and isoform B (IR-B) that includes exon 11. A number of studies began to report different characteristics of the two isoforms: Mosthaf et al. observed that when the cDNAs encoding the two isoforms were expressed in Rat1 cells, IR-A exhibited a higher affinity for insulin than the IR-B isoform (3). Yamaguchi et al. reported differential ligand binding to the two isoforms: labeled-insulin had a faster “on and off rate” for IR-A compared with IR-B (4, 5). These data suggested that the isoforms may perform slightly different functions as receptors for insulin but potentially much more profound differences became clear when Frasca et al. determined that IR-A and not IR-B acted as a high-affinity receptor for insulin-like growth factor-II (IGF-II) (6). Along with IGF-I, IGF-II is a peptide belonging to the IGF family that in addition to the two ligands, also comprise six high-affinity IGF binding proteins (IGFBPs), IGFBP-proteases, together with the IGF-I and IR receptors and a non-signaling IGF-IIR, that acts to clear excess IGF-II: components of this system are often dysregulated in a number of different cancers, including that of the prostate (7). Prostate cancer (PCa) is the fifth most common cause of cancer death worldwide for males, and the eighth most common cause of cancer death overall. It is predicted that there will be 27,540 deaths from PCa in the United States in 2015 (American Cancer Society: http://www.cancer.org/cancer/prostatecancer). The major issue in the management of PCa is the inability to distinguish between the majority that are slow growing and localized, prostate tumors from the less common but more invasive, aggressive, and metastatic PCas that will impact on mens’ lives. Whereas cancers localized to the prostate are effectively cured through surgery or radiotherapy, few treatments are available for men with metastatic or hormone-therapy resistant PCa (8). Population studies have clearly implicated metabolic factors (IGFs/insulin) as contributors to disease progression and poor response to therapy (9). Both IGF-I and IR receptors are present on human PCas (10) and as with other cancers, it has been reported that the IR-A as opposed to the IR-B isoform is predominant in the cancer tissue compared to levels observed in the benign prostate samples (11). In this study, we assessed alterations in the IR-A and IR-B isoform ratio and associated changes in cell proliferation and migration in PCa cell lines following exposure to altered concentrations of glucose and treatment with IGF-II and insulin. We also examined the abundance of the IR isoforms in areas of benign tissue and invasive prostate carcinoma from the same patient in a small cohort of PCa patients diagnosed between 2000 and 2009 with clinically localized hormone-naïve adenocarcinoma of the prostate.

## Materials and Methods

### Materials

All chemicals, unless otherwise stated, were purchased from Sigma (Gillingham, UK). All siRNAs and the transfection reagent, Saint-Red were purchased from Synvolux Therapeutics B.V. (Groningen, The Netherlands). Fetal bovine serum (FBS) was purchased from Invitrogen (Paisley, UK), DMEM-25 mM glucose (BF-709) and DMEM-5 mM glucose (BF-708), RPMI 1640, penicillin–streptomycin solution and l-glutamine were bought from Lonza (Basel, Switzerland). Human, recombinant IGF-II was obtained from GroPep (Thebarton, Australia) and Actrapid (human insulin) was purchased via Southmead Hospital Pharmacy.

### Cell Culture

We used PCa cell lines: androgen-independent DU145, PC3 and androgen-dependent LNCaP and VCaP that were purchased from the American Type Culture Collection (ATCC) that authenticates their cell lines using short tandem repeat (STR) DNA profiles; the cells were used for a maximum of 10 passages. DU145, PC3, and VCaP cells were grown in DMEM growth media (GM) supplemented with 10% FBS, penicillin–streptomycin (50 IU/ml) and 1% l-glutamine solution (2 mM) and LNCaP cells were cultured in RPMI 1640 GM supplemented with 10% FBS and antibiotics. Cells were maintained in a humidified 5% carbon dioxide atmosphere at 37°C. For all experiments, cell lines were seeded in 5 mM glucose GM for 24 h and then transferred to either serum-free media (SFM); supplemented with sodium bicarbonate (1 mg/ml), bovine serum albumin (0.2 mg/ml), and transferrin (0.01 mg/ml) for a further 24 h.

### Cell Counting

Floating cells were collected and mixed with adherent cells after trypsinization, and the resulting cell suspension was loaded onto a hemocytometer (1:1) with the dye Trypan blue, which is taken up by dead cells. Both viable and dead cells were counted, from which both the percentage of dead cells and total cell number were calculated.

### Real-Time Polymerase Chain Reaction and Quantitative Polymerase Chain Reaction

Real-time polymerase chain reaction (RT-PCR) and quantitative polymerase chain reaction (q-PCR) were used to analyze the expression of the IR and the IR isoforms. Total RNA was extracted using Trizol reagent (Invitrogen, Carlsbad, CA, USA) according to the manufacturer’s instructions. Two micrograms of total RNA was used for cDNA synthesis with random hexamers. RNA was quantified using a Nano photometer (used at a 260/280 ratio of between 1.6 and 2.0). For RT-PCR, primers for detecting both IR-A and IR-B were designed “in-house”: forward 5′ GGAAGACGTTTGAGGATTAC 3′ and reverse 5′ TCGAGGAAGTGTTGGGGAAAG3′. PCR was carried out using HotStarTaq DNA polymerase (Qiagen, Manchester, UK) per the manufacturer’s recommendations. In brief, the PCR program was run at 95°C (5 min) [95°C (1 min), 59°C (1 min), and 72°C (1 min) × 40] and 72°C (10 min). PCR products were run on a 2% agarose gel and detected using an ultraviolet transilluminator (Biorad Gel Doc 1000, Hertfordshire, UK). To verify the IR-A and B PCR products, they were commercially sequenced (Source BioScience, Nottingham, UK) and were verified to be the sequence listed in GENE BANK (NM_000208.2). Quantitative real-time PCR was performed using Green JumpStart SYBR and an ABI StepOne Plus Realtime PCR System (Applied Biosystems; Life Technologies, Paisley, UK). In brief, the PCR program was run at 95°C (2 min) followed by (95°C (15 s) and 60°C (30 s) × 40). Reactions were run in duplicate in three independent experiments. Expression data were normalized to the geometric mean of a housekeeping gene (18S) to control the variability in expression levels and were analyzed using the 2^−ΔΔCT^ method. Melting curve analysis was performed each time to confirm specific PCR amplification. PCR primers were designed using OligoPerfect online software from Qiagen (Manchester, UK) under consideration of the special design criteria for real-time RT-PCR primers, spanning the junction between exons. For IR forward 5′TGACAACGACCAGTGTGGAG 3′ and IR reverse 5′ GCAGCCGTGTGACTTACAGA 3′, for 18S forward 5′GATGTAGTTGCTTGGGACCCA 3′ and 18S reverse 5′TGGAGATAACACTCTAAGCATAACTAAAGGT 3′.

#### Transfection of Cells with siRNA

Cells were transfected with siRNA using Saint-Red following the manufacturer’s instructions and as described previously (12). IR-A and IR-B were knocked down using specifically designed siRNAs that spanned exon 11 to 12 of the IR (for IR-B silencing) and exon 10 to12 (for IR-A silencing): target sequence IR-A; UUUCCGAGAUGGCCUGGGGUU. IR-B; CGAGGACCCUAGGCCAUCUUU. A non-silencing (NS) negative control siRNA was used as a second control.

#### Migration Assay

A cell suspension of DU145 cells (70 μl: 0.5 × 10^4^ cells/ml of 5 mM GM) was placed into each chamber of an Ibidi Culture-Insert (Thistle Scientific Ltd, Glasgow, UK) in a well of a 6-well plate. After cells had attached, reached confluency in the chambers (100%) and formed a tight junctioned gap, the culture insert was gently removed and cells were exposed to either 5 or 25 mM SFM in the presence or absence of IGF-II or insulin (each at 100 ng/ml) for the times denoted in the figure legend. Microscopy images (Leica DM IRB, Milton Keynes, UK) were assessed using Image J to measure changes in the width of the gap.

#### Western Blotting

Cell lysates were run on an 8% SDS-PAGE and transferred to Hybond-C nitrocellulose membrane (GE Healthcare, Bucks, UK). Proteins were probed with anti-IR (1:500 Santa Cruz, Heidelberg, Germany) anti-GAPDH or anti-β actin (Millipore, Hertfordshire, UK, both 1:10,000), anti-IR-β (1:750 Santa Cruz, Heidelberg, Germany), anti-p-Akt, anti-Akt, anti-p-MAPK and anti-IGF-IR (all 1:1000), and anti-MAPK (1:500) and anti-p-IGF-IR (1:750) (all from Cell Signalling, Leiden, The Netherlands) with secondary antibodies as follows: anti-rabbit (1:2500; IR, IR-β, p-Akt, Akt, p-MAPK, MAPK, p-IGF-IR, and IGF-IR all at 1 :2000) and anti-mouse (1:10,000 for both GAPDH and β-actin). All antibodies were diluted following the manufacturer’s instructions. Proteins were visualized using supersignal west dura ECL solution (Thermo Fischer, Ulm, Germany) and the UVP ChemiDoc-IT Imaging System (UVP, Bio-Rad, Hertfordshire, UK) and analyzed using Vision Works ls Analysis Software (UVP Inc., Upland, CA, USA).

### Human Tissue Samples

This study used tissue samples from 37 PCa patients diagnosed between 2000 and 2009 with clinically localized hormone-naïve adenocarcinoma of the prostate. The formalin fixed paraffin embedded (FFPE) PCa samples under approval of National Research Ethics Committee (NRES No. 09/H0102/48) were kindly assessed in collaboration with Dr. Rachel Hagen (University of the West of England, Bristol, UK). Tissue samples were fixed in neutral buffered saline for at least 24 h before processing and the wax blocks were stored at room temperature. Three consecutive 10-μm wax curls were cut from each sample case. An FFPE RNeasy Kit (Qiagen, Manchester, UK) was used to isolate tissue RNA; each wax curl was placed in a 1.5 ml Eppendorf and 1 ml of Histoclear was used to deparaffinise at 56 for 5 min. Tissue was pelleted by centrifugation and the Histoclear removed; the pellet was washed twice with 70% ethanol. After 10–15 min of air-drying, lysis buffer containing proteinase K (from kit) was added to the sample that was then incubated at 56 for 16 h. After proteinase K digestion the sample was incubated at 80 for 15 min and placed on ice for another 3 min. Samples were centrifuged and the supernatant added to 1 ml of Trizol reagent followed by the standard RNA isolation and RT-PCR protocol described in RT and q-PCR section above to assess the expression of the IR-A and B isoforms.

### Statistical Analysis

Data show the mean SEM. Data were analyzed with SPSS 12.0.1 for Windows using one-way ANOVA followed by least significant difference *post hoc* test. A statistically significant difference was considered to be present at *p* < 0.05.

## Results

### Insulin Receptor Isoform Profile in Prostate Cancer Cell Lines

**Figure 1:**
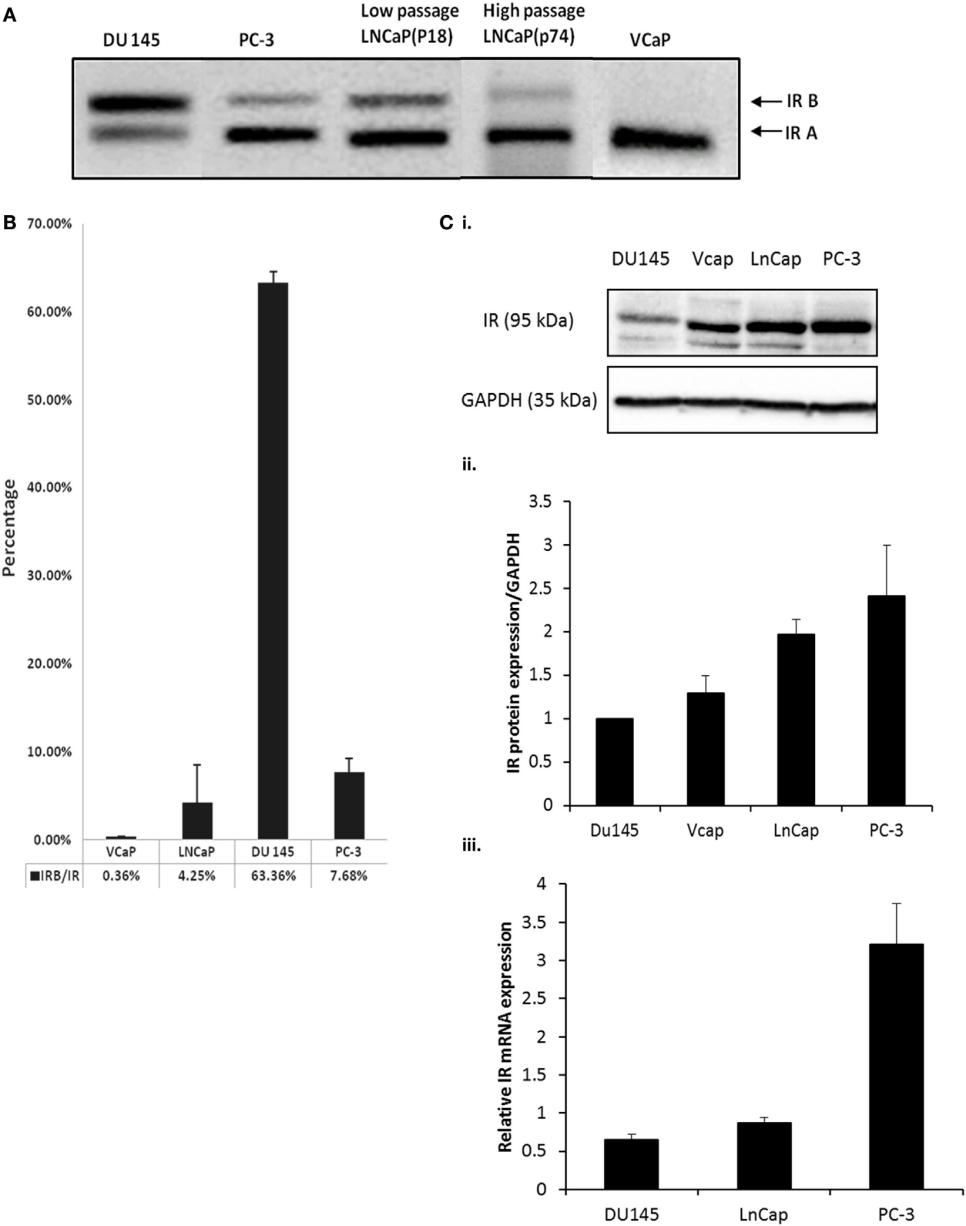
**(A)** DU145, PC-3, LNCaP (low passage P18 and high passage P74) and VCaP cells were cultured in GM for 72 h followed by RNA extraction and IR-A and IR-B assessment detected by RT-PCR. The size of IR-B and IR-A were 171bp and 135bp, respectively. **(B)** q-PCR was used to detect the percentage of IR-B expression compared to total IR expression in different cell lines. IR-B, IR, and reference gene 18s primer pairs were used to amplify the signal of each target gene. The graph shows the percentage of IR-B compared to IR: the error bars represent the SE of the mean of the repeats and each experiment was performed in triplicate (*n* = 3). **(C) (i)** Western immunoblot of whole cell lysates extracted from DU145, PC-3, LNCaP, and VCaP cells after the cells had reached a confluence of 80% in GM to show the abundance of the IR relative to GAPDH used as a loading control. This is representative of blots performed at least three times. The optical density measurements showing the mean of the three repeats are shown in **(C) (ii)**. **(C) (iii)** shows q-PCR of mRNA levels for the IR in DU145, LNCaP, and PC3 cells (*n* = 3).

Using RT-PCR, we observed that PC3, LNCaP (low and high passage) and VCaP cells each expressed less of the IR-B isoform compared to IR-A. By contrast, the DU145 cell line exhibited the opposite profile having more IR-B than the IRA isoform (Figure 1A). q-PCR confirmed these observations by assessing the percentage of the IR-B isoform compared to the total amount of the IR. DU145 cells exhibited the highest IR-B expression (63.4%), while PC-3 and LNCaP cells predominantly expressed IR-A over IR-B (7.7 and 4.3%, respectively, of IR-B) and VCaP cells had almost no signal for IR-B (0.4%) (Figure 1B). Each of the cell lines expressed the IR with abundance being higher in the PC-3, VCaP, and LNCaP cells compared with the DU145 cells with mRNA levels also being lowest in DU145 and highest in PC3 cells (Figures 1Ci,ii,iii).

### Manipulation of the IR Isoforms

#### siRNA to Silence the IR-A and IR-B Isoforms

**Figure 2:**
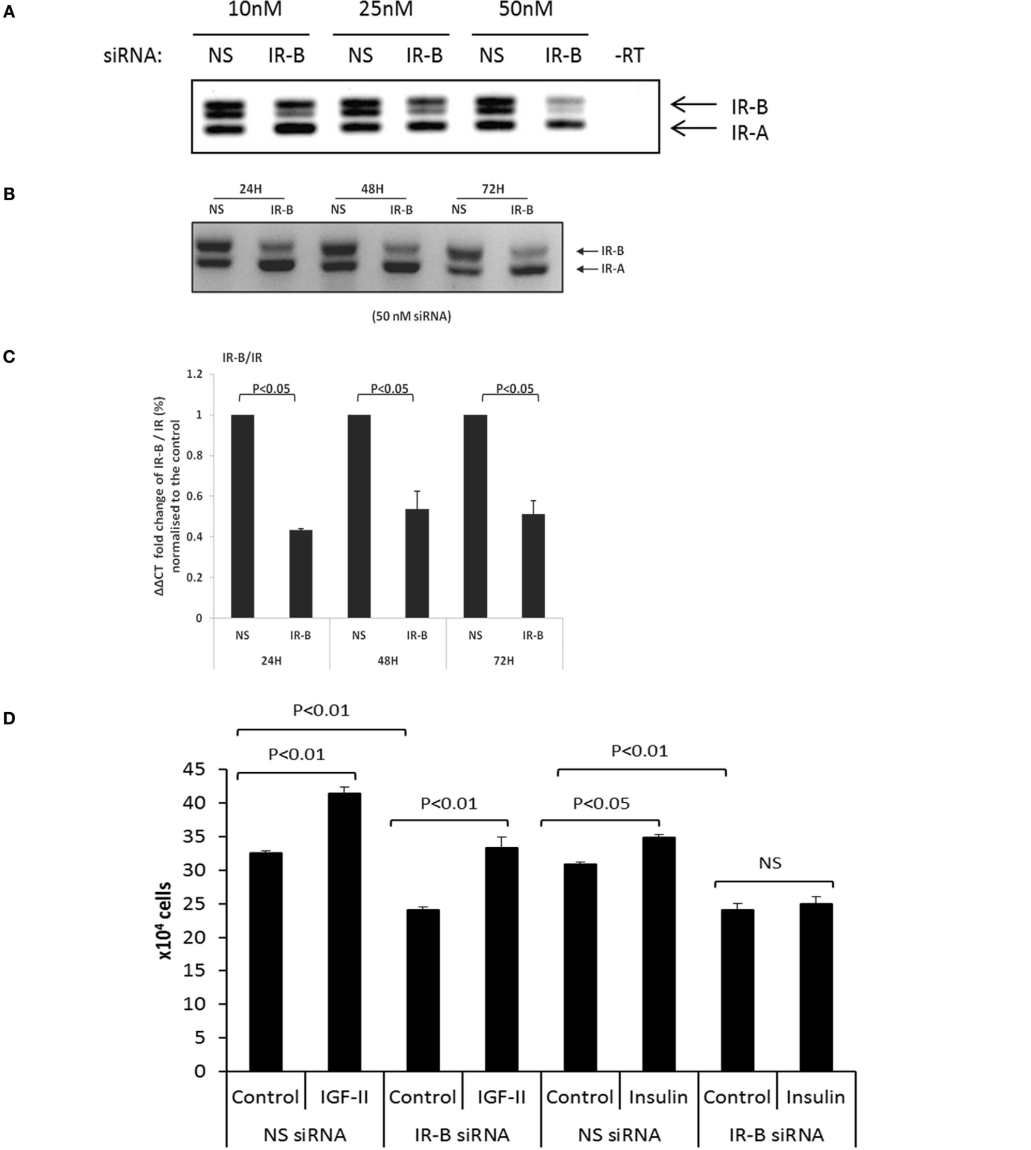

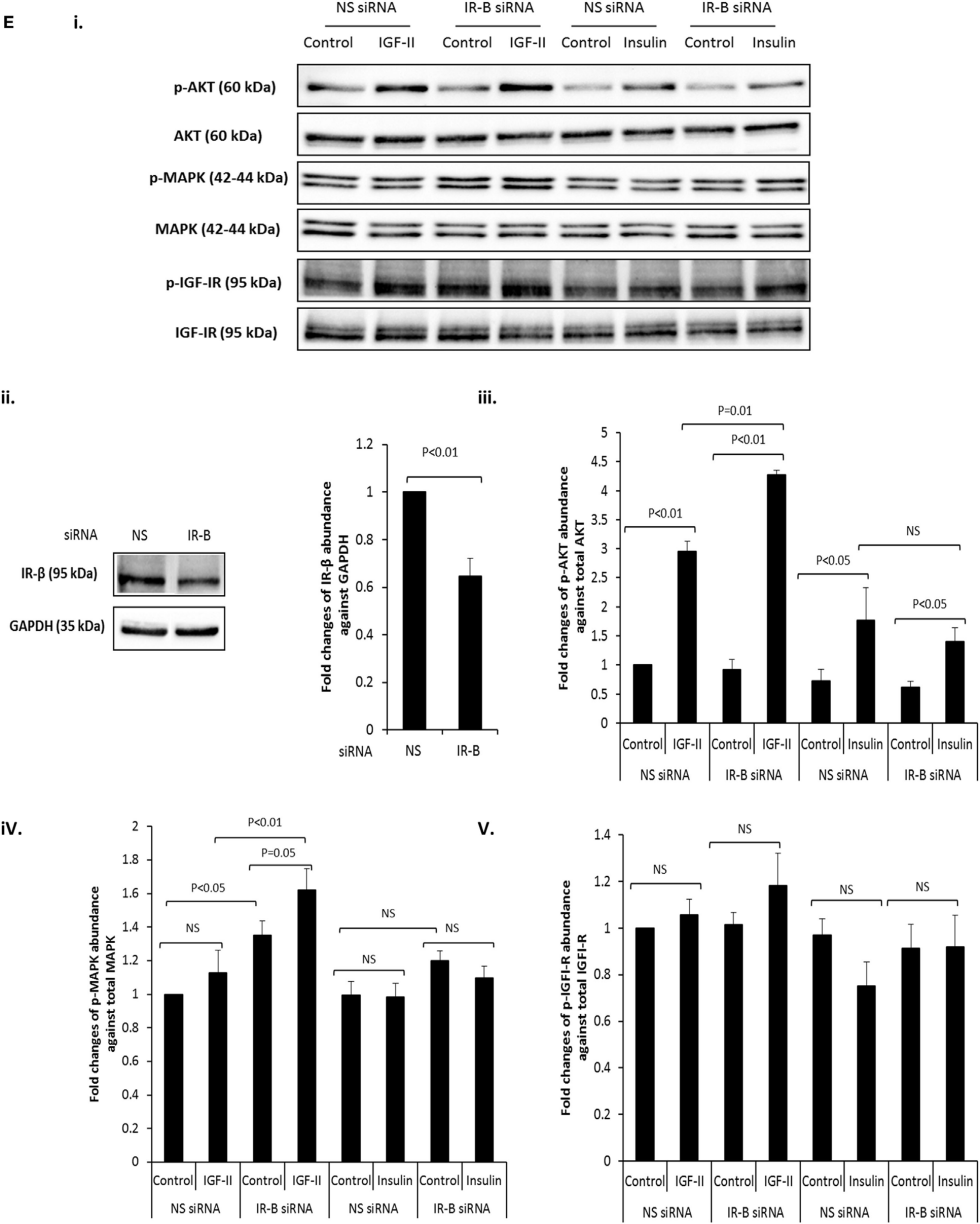
**(A)** RT-PCR blot in which siRNA (10, 25, and 50 nM) was used to specifically silence IR-B in DU145 cells. Cells treated with a non-silencing (NS) siRNA acting as a negative control. After 24 h, sample RNA was extracted and RT-PCR was used to assess the expression of the IR-B isoform. This is representative of blots performed at least three times. **(B)** Representative RT-PCR blot shows the effect of 50 nM IR-B siRNA in DU145 cells after 24, 48, and 72 h of treatment. A non-silencing siRNA acted as a negative control. This is representative of blots performed at least three times. **(C)** Q-PCR ΔΔCT IR-B/IR fold expression (%) normalized to non-silencing samples. IR-B was silenced for 24, 48, and 72 h in DU145 cells. Error bars represent the SE of the mean from three experiments each repeated in duplicate (*n* = 3). **(D)** DU145 cells were seeded in six well plates (0.1 × 10^6^ cells per well) in the presence or absence of target siRNA to the IR-B or non-silencing (NS) siRNA for 24 h and then switched to serum-free media for a further 24 h followed by dosing with IGF-II or insulin for 48 h. Changes in cell proliferation were assessed by direct count of viable cells in a hemocytometer. Results shown are the mean of three independent experiments each repeated in triplicate. Data are represented as mean ± SEM. **(E) (i)** DU145 cells were seeded in 6 well plates (0.1 × 10^6^ cells per well) in the presence or absence of target siRNA to the IR-B or non-silencing (NS) siRNA for 24 h and then switched to serum-free media for a further 24 h followed by dosing with IGF-II or insulin for 30 min. Western blotting was performed to show protein abundance of p-AKT/AKT, p-MAPK/MAPK, and p-IGF-IR/IGF-IR (*n* = 3 experiments). **(ii)** shows a western blot and optical density measurements for levels of IR-β following IR-B silencing and **(iii–v)** shows the mean (*n* = 3) optical densitometry measurements from the western blots for p-AKT/AKT, p-MAPK/MAPK, and p-IGF-IR/IGF-IR respectively: a representative example of which is shown in **(E) (i)**.

Using RT-PCR with increasing concentrations of the IR-B siRNA, we observed effective silencing in DU145 cells with 50 nm siRNA for 24 h and this was sustained for 72 h (Figures 2A–C). q-PCR confirmed effective silencing of the IR-B isoform at each time point (24, 48, and 72 h; *p* < 0.05, respectively) and also showed that a reduction in the IR-B isoform relative to the IR or the reference gene (18S rRNA) was not associated with a reduction in absolute levels of the IR mRNA or protein up to 72 h (Figure S1A,B in Supplementary Material). We were unable to silence the IR-A isoform in DU145 cells despite varying the concentration of the siRNA or the exposure time (data not shown). In the other three PCa cell lines that we investigated (LNCaP, PC3, or VCaP), all express less than 10% of their IR in the IR-B isoform, we were unable to silence either IR-A or IR-B. However, using the IR-B siRNA that worked in the DU145 cells we were able to also effectively silence IR-B in a breast cancer cell line (Figure S1C in Supplementary Material). We found that silencing the IR-B isoform in the DU145 cells resulted in a significant decrease in basal cell proliferation as measured by cell counting (*p* < 0.01; Figure 2D). Addition of 25 ng/ml of IGF-II or insulin alone to the DU145 cells significantly increased cell proliferation (*p* < 0.01 and *p* < 0.05, respectively), with IGF-II having the greatest effect. Silencing the IR-B isoform did not affect the response to IGF-II, whereas the response to insulin was blocked (Figure 2D). We also assessed the acute signaling response to IGF-II and insulin in the presence or absence of siRNA to IR-B. We showed effective silencing of IR-B using RT-PCR (Figures 2A–C) and Figure 2Eii indicates that protein levels of IR-β were also reduced. With Akt activation, we found that IR-B knockdown alone had no effect. We observed that after 30 min of treatment IGF-II and insulin each induced a significant increase in Akt activation that was unaffected when IR-B was silenced. There were no significant effects of either IGF-II or insulin in the presence or absence of siRNA to IR-B on p-MAPK or p-IGF-IR. We did, however, notice that with IR-B silenced alone there was a suggestion that MAPK was activated.

#### Exposure to Varying Concentrations of Glucose

**Figure 3:**
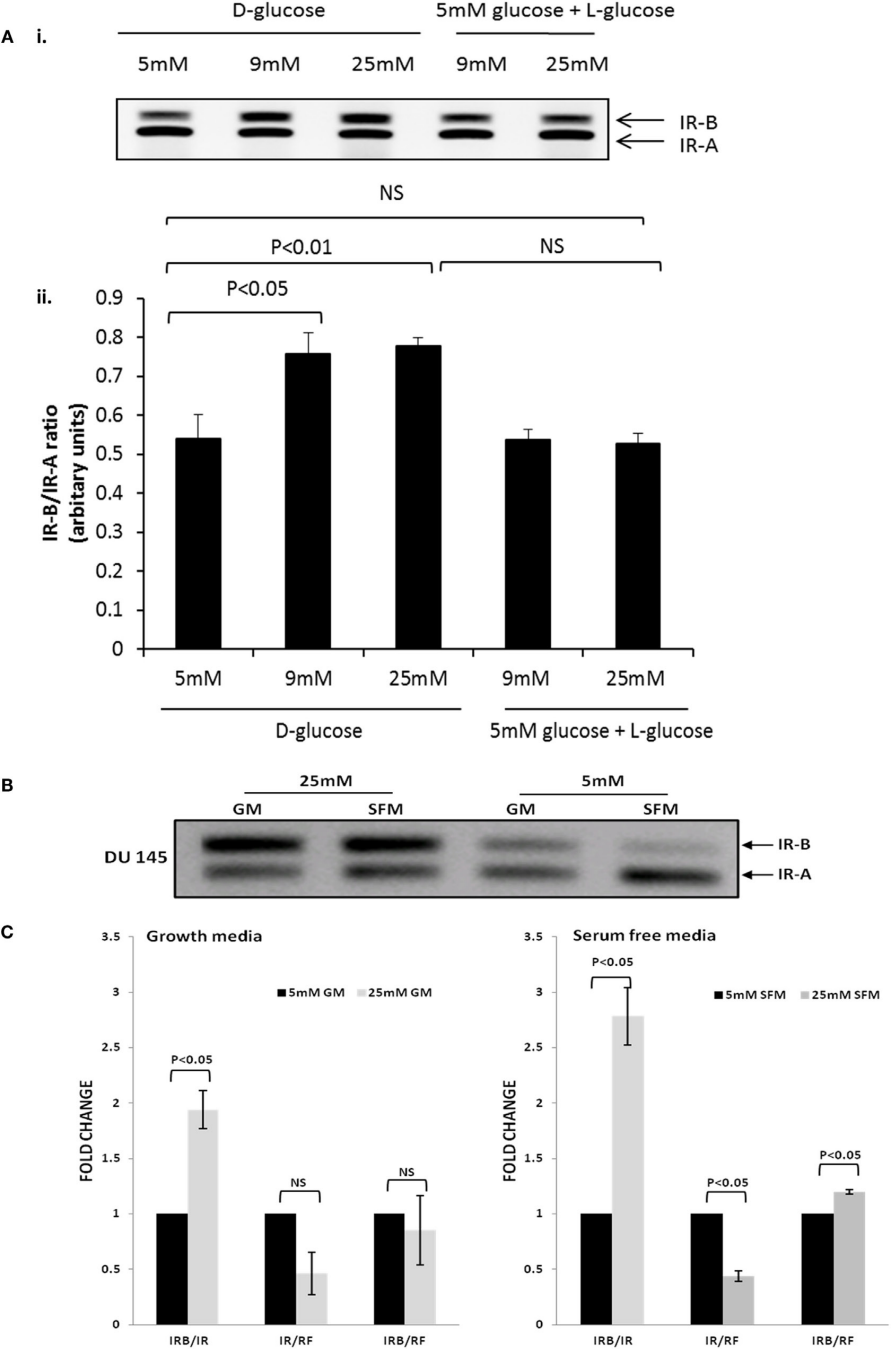

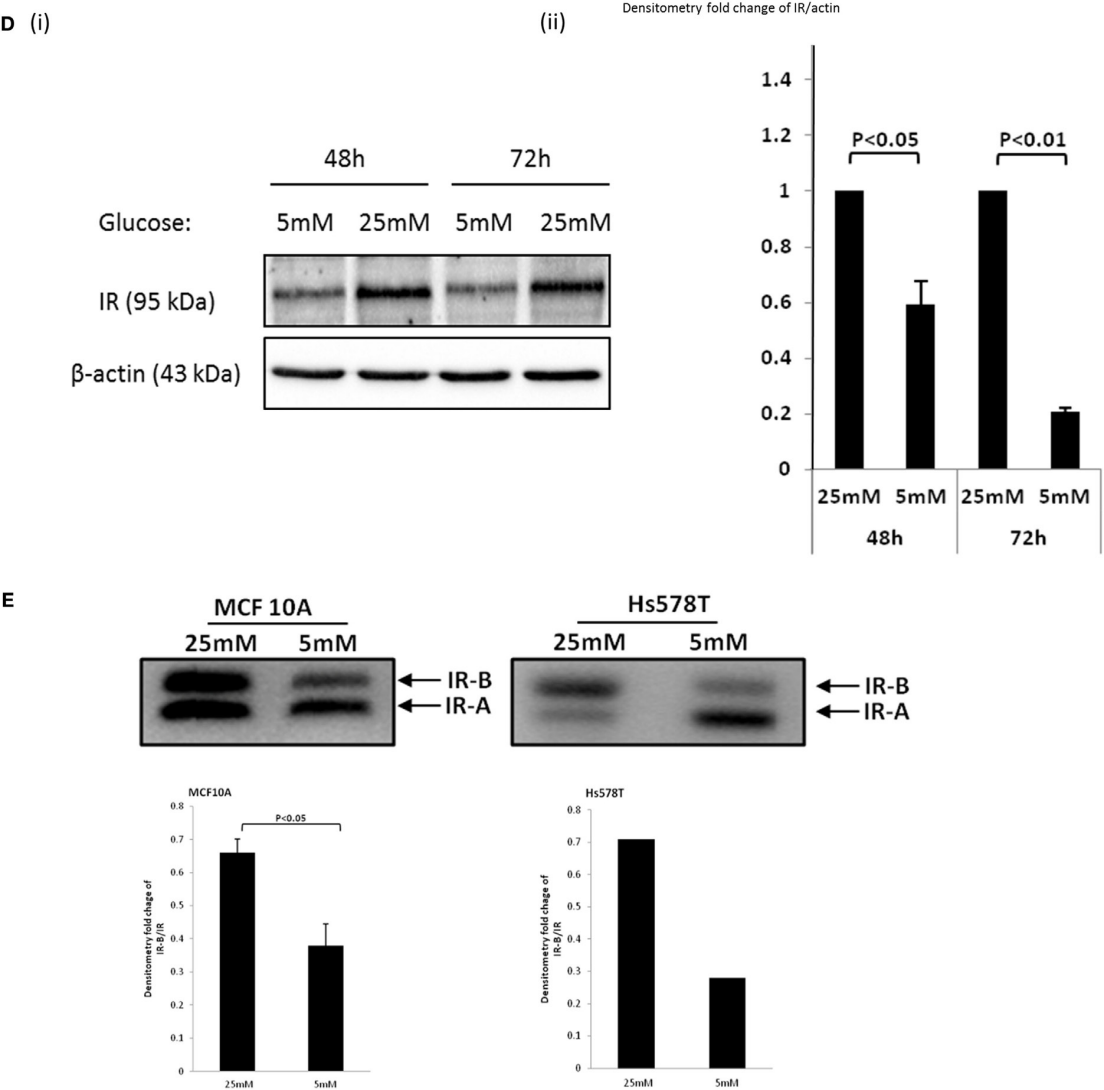
**DU145 cells were seeded in T25 flasks (0.6 × 10^6^ cells) in 5 mM d-glucose containing GM**. After 24 h, the media was changed to 5, 9, and 25 mM d-glucose or 5 mM d-glucose supplemented to 9 and 25 mM with l-glucose-containing GM for a further 48 h. RNA was extracted and reversed to cDNA. **(A) (i,ii)** RT-PCR analysis to assess the expression of the IR isoforms and the associated arbitrary units indicating the changes in the ratio of the IR isoforms respectively. DU145 cells were seeded in T25 flasks (0.6 × 10^6^ cells) in 5 mM GM. After 24 h, the media was changed to 25 or 5 mM glucose-containing GM or SFM, respectively, for a further 48 h. RNA was extracted and reversed to cDNA. **(B)** RT-PCR analysis to assess the expression of the IR isoforms. **(C)** q-PCR data comparing fold change of the relative IR-B gene expression compared to the IR or housekeeping gene 18s (RF). Error bars represent the SE of the mean from three experiments each repeated in duplicate (*n* = 3). **(D) (i)** A representative western blot to show IR protein abundance in DU145 cells after being placed in 25 or 5 mM glucose conditions for different time points. Protein was extracted after 48 and 72 h and detected by western blotting using an IR (95 kDa) antibody. Housekeeping protein β-actin (43 kDa) was used as loading control. **(D) (ii)** graph showing fold changes in densitometry for IR protein relative to loading control. Error bars represent the SE of the mean of three repeats (*n* = 3). **(E)** A representative RT-PCR blot of IR isoform expression in MCF10A and Hs578T cells after 48 h exposure in 25 and 5 mM glucose SFM after being seeded for 24 h in 5 mM glucose GM. Relative OD ratio of IR-B isoforms in the MCF10A cell line (*n* = 3). Relative OD ratio of IR-B isoforms in Hs578T cells (*n* = 1).

We performed a dose response and exposed the DU145 cells to increasing levels of glucose (5, 9, and 25 mM). Using RT-PCR, we found that the ratio of IR-B to IR-A increased in 9 mM that was not increased further with 25 mM concentrations of glucose (Figure 3Ai,ii). We further demonstrated that the effect of glucose on increasing the IRB:IR-A ratio was specific and not due to changes in osmolarity as exposing the cells to 5 mM d-glucose supplemented to 9 and 25 mM with l-glucose (that the cells are unable to metabolize) had no effect (Figure 3Ai,ii). We then performed a time course and exposed the DU145 cells to normal (5 mM) or high (25 mM; concentrations routinely used in cell culture) levels of glucose in SFM for up to 72 h. Using RT-PCR, we found that the ratio of IR-B to IR-A increased when the cells were exposed to high glucose conditions (as we observed in Figure 3A): this occurred from the earliest time point of 6 h and was maintained for up to 72 h (data not shown). RT-PCR data confirmed that after 48 h, the IR-B mRNA expression was upregulated significantly in 25 mM glucose conditions in both GM and SFM (Figure 3B). q-PCR was used to measure the quantitative fold change of the IR-B isoform compared to the IR at 48 h: the IR-B isoform increased 1.9-fold (in GM) and 2.8-fold (in SFM) compared to total IR mRNA under hyperglycemic conditions. We also showed that the effect of the high glucose in increasing the IR-B isoform was reversible when the cells were returned to normal glucose media (Figure S1D in Supplementary Material). In addition, absolute IR-B and total IR message levels, compared to the reference gene, did not change significantly in GM, whereas in SFM, levels of absolute IR-B were decreased (*p* < 0.05) and absolute levels of IR were increased (*p* < 0.05) in normal compared to hyperglycemic conditions (Figure 3C). Using western immunoblotting, we observed that total levels of the IR protein were significantly reduced (*p* < 0.05) in SFM containing 5 mM compared to 25 mM glucose after 48 h that was sustained for 72 h (Figure 3Di,ii), which did not mirror the changes observed at the mRNA level. We did not observe this hyperglycemia-induced increase in the IR-B isoform with LNCaP, PC3, or VCaP cells, that all inherently express predominantly IR-A (Figures S1E–G in Supplementary Material); but a similar effect was observed both in normal breast epithelial cells (MCF-10A) and in the HS578T breast cancer cell line (Figure 3E).

#### Exposure to Varying Concentrations of Glucose in the Presence of IGF-II and Insulin

**Figure 4:**
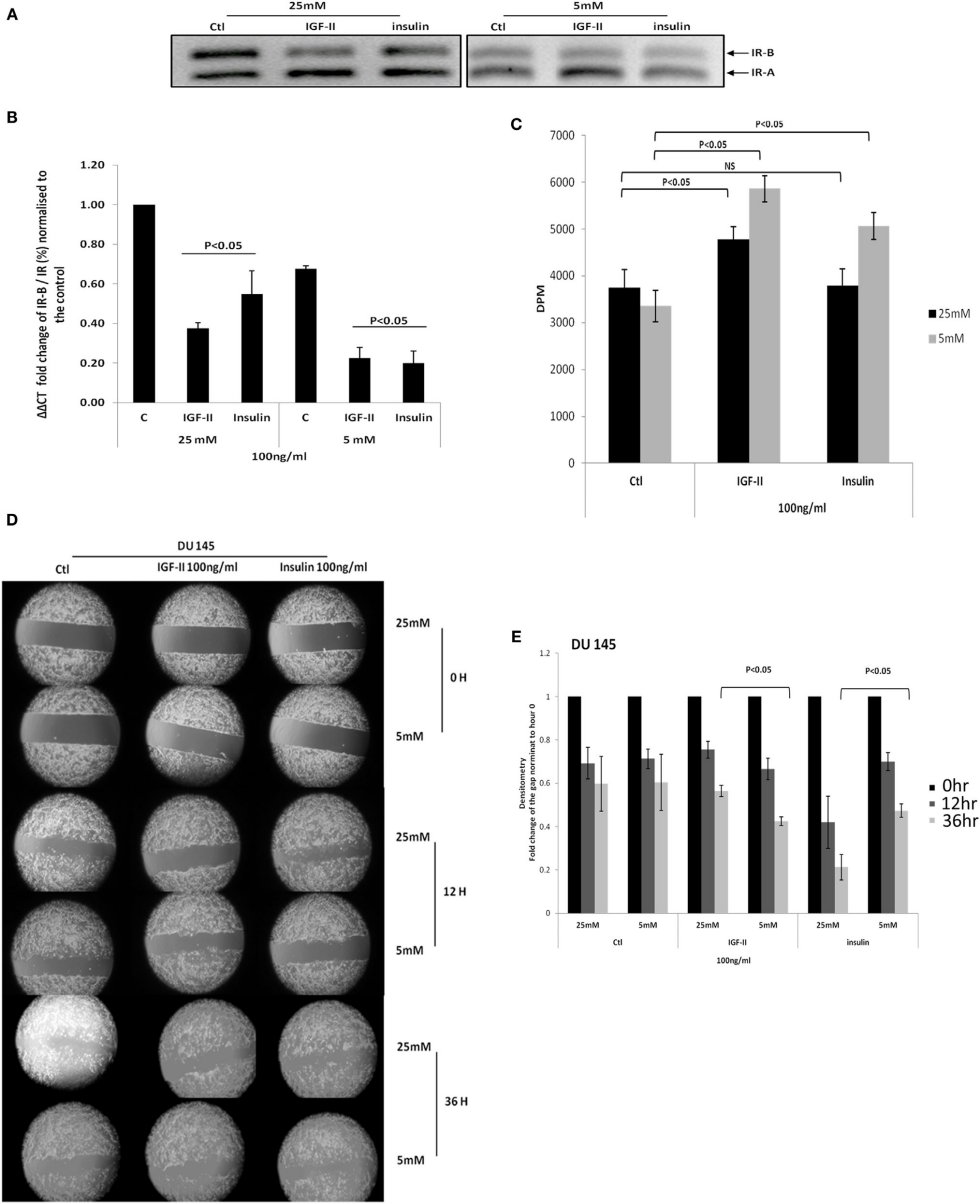
**(A)** DU 145 cells were seeded at 0.3 × 10^6^ cells per T25 flask in 5 mM glucose GM for 24 h before being changed to 25 or 5 mM glucose-containing SFM conditions for a further 24 h. Cells were then treated with 100 ng/ml IGF-II or insulin for 48 h: mRNA was analysis by RT-PCR. **(B)** The fold change in IR-B/IR was assessed by q-PCR. Error bars represent the standard error of the mean from three experiments each repeated in duplicate (*n* = 3). **(C)** DU145 were seeded in 24 well plates (0.0110^6^ per well) in 5 mM glucose-containing GM for 24 h and then changed to either 5 or 25 mM glucose-containing SFM for 48 h, followed by dosing with either 100 ng/ml of IGF-II or insulin for another 72 h. Proliferative activity was detected by TTI assay. Error bars represents the SE of the mean from five experiments each repeated in triplicate (*n* = 5). **(D)** DU145 cells were seeded into culture-inserts in 6 well plates in 5 mM GM. After 24 h, cells were treated with either 100 ng/ml IGF-II or insulin in either 25 mM or 5 mM-glucose containing SFM. Pictures were taken at time 0, 12, and 36 h. These are representative of experiments repeated three times. **(E)** Densitometry shows the fold change of the gap relative to time 0. Error bars represent the SE of the mean of three repeats (*n* = 3).

Figure 4A confirms the data in Figure 3 showing that with DU145 cells high glucose compared to normal glucose conditions increases the ratio of the IR-B to IR-A isoform. In addition, using q-PCR, we confirmed that the IR-B isoform was increased relative to the absolute levels of the IR mRNA under hyperglycemic conditions and that, in both concentrations of glucose, IGF-II and insulin each down-regulated the IR-B isoform compared to their respective controls (*p* < 0.05, respectively; Figure 4B). This shows that in normal glucose conditions in the presence or absence of insulin/IGF-II, the predominant isoform appears to be IR-A. Under hyperglycemic conditions, IR-B is the predominant isoform, but clearly on exposure to IGF-II/insulin the isoform ratio can be reverted back to mainly IR-A. Figure 4C shows that basal cell proliferation was unaffected by altering glucose concentrations. However, increasing levels of glucose negated the ability of insulin to increase proliferation and reduced the proliferative response to IGF-II (Figure 4C) consistent with the observed proliferative effects of insulin and IGF-II in high glucose that were observed in Figure 2D. In high glucose, when the proportion of IR-B was increased, the changes in response to insulin and IGF-II were the opposite to those observed when the proportion of IR-B was reduced by siRNA. Figures 4D,E indicate that migration in treated and untreated samples was evident at 12 h and basal migration was unaffected by altering levels of glucose. By 36 h IGF-II induced more migration in normal compared to high glucose (*p* < 0.05): consistent with a greater response to IGF-II when there was more IR-A compared to IR-B, whereas the opposite was observed for insulin: cells responded better to insulin in high compared to normal levels of glucose (*p* < 0.05): consistent with a greater response to insulin when there was an increase in IR-B compared with IR-A.

### Insulin Receptor Isoform Profile in Prostate Tissue Samples

**Figure 5:**
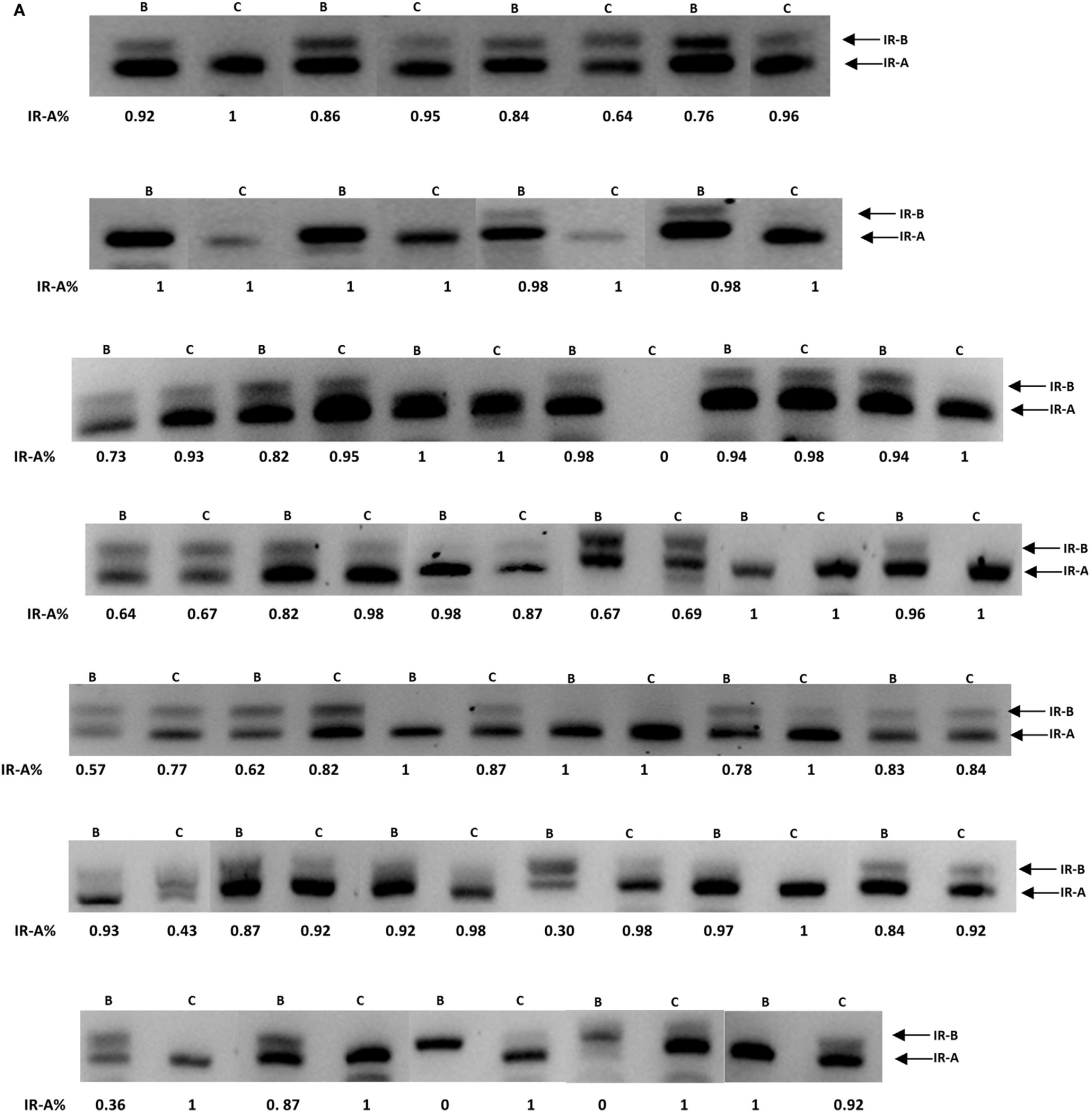

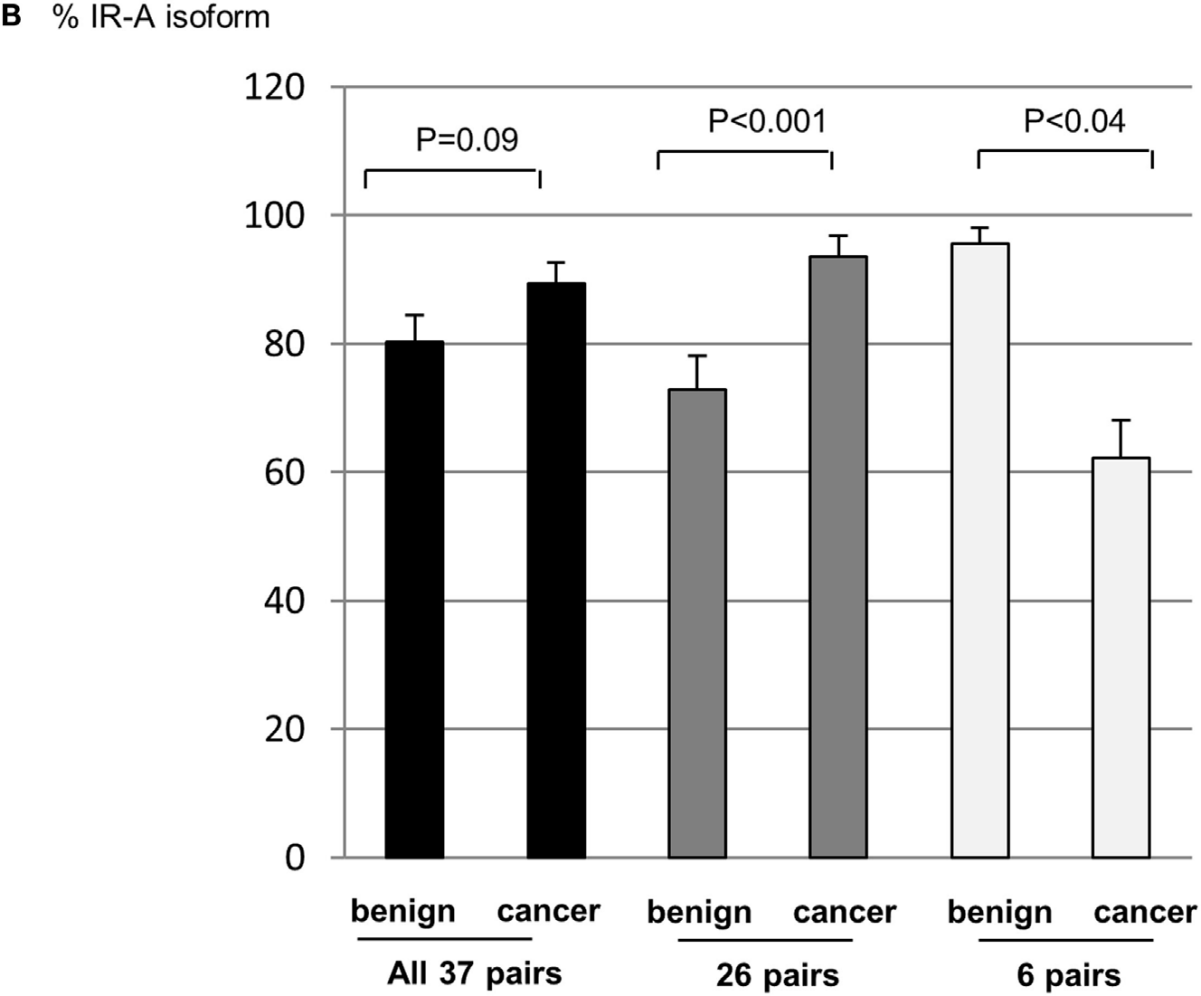
**(A)** FFPE patient samples were collected from patients diagnosed with adenocarcinoma of the prostate. RT-PCR was used to assess the levels of the IR-A and -B isoforms. **(B)** Graph showing the percentage change in the IR-A isoform relative to IR-B. Error bars represent the SE of the mean, where *p* < 0.05 represents statistical significance. B, benign; C, cancer.

We assessed the overall difference in expression of the isoforms in the 37 pairs of samples (benign and cancer) and found that the IR-A isoform tended overall to be greater by 9.1% (*p* = 0.09) in the cancerous regions of the samples compared to the benign regions. There was considerable heterogeneity between samples with the majority of samples exhibiting predominantly the IR-A isoform in both benign and cancerous regions. There were, however, exceptions where the IR-B isoform predominated. The IR-A isoform was greater in the cancer, compared to benign regions in 26 pairs (by 20.8%; *p* < 0.001). Only six pairs showed a reduced proportion of the IR-A isoform in the cancer regions (by 33.4%: *p* < 0.05) with no difference in expression observed in five pairs (Figures 5A,B).

## Discussion

Men who develop PCa increasingly have one of the co-morbidities associated with a Western lifestyle (obesity, metabolic syndrome or diabetes). These co-morbidities are characterized by hyperinsulinemia, hyperglycemia, and increased expression of IGF-I and -II that have each been associated with poor prognosis and more aggressive cancers that exhibit increased metabolism and increased glucose uptake (13–15). The IR has two isoforms IR-A and IR-B: IR-A has a higher affinity for IGF-II comparable to that for insulin, whereas the IR-B isoform predominantly binds to insulin.

It has been reported previously that the IR-A isoform is upregulated in a number of different tumor types, including those of the breast, colon, and lung (6). Despite assessing changes in a relatively small cohort of 37 PCa patients, we observed similar changes: of the 37 paired samples the IR-A isoform was greater overall in the cancerous regions of the samples compared to the benign regions adjacent to the tumor tissue and was the predominant isoform in the cancer compared to benign regions in 26 pairs. This is consistent with a previous study that examined tissue from 30 samples (11). We noticed considerable heterogeneity between samples with the IR-A being predominant in the benign tissue of some pairs and exceptions where the IR-B isoform predominated. Heni et al. compared the IR isoform ratio, in histologically benign prostatic tissue adjacent to the primary malignant cancer. The authors reported that the IR-A isoform was predominant in PCa tissue and was reduced in benign tissue adjacent to the tumor tissue, but that this difference in isoform expression between tumor and benign tissue was only significant when comparing tumor tissue to tissue derived from an entirely benign prostate (11). Prostatic intraepithelial neoplasia (PIN) describes dysplastic changes in prostate cells that line the prostate gland and is considered to be a pre-malignancy, or carcinoma *in situ* of the prostate gland. PIN coexists with cancer in the majority of cases, but retains an intact or fragmented basal cell layer, unlike cancer, which lacks a basal cell layer (16). As the benign regions we studied were adjacent to tumor tissue in the same sample, it is possible that for some cases areas of PIN were included which may explain why some of our patient samples had more IR-A than IR-B in the benign region. Another, more interesting, possibility relates to reports of the common occurrence of loss of imprinting of the IGF-II gene in PCas (17) that has been described as a field effect throughout the prostate (18). We have shown that exposure of PCa cells to IGF-II results in a suppression of the IR-B isoform and, therefore, increased expression of IGF-II, due to loss of imprinting throughout the prostate could result in suppressed IR-B and the predominance of IR-A throughout the prostate. An examination of benign prostate samples from cases of benign prostatic hypertrophy (BPH) that are cancer free compared with cancers, with and without the loss of imprinting of IGF-II, would be required to establish this point. Importantly, since IR-A and R-B can only be distinguished by RT-PCR from total tissue RNA extract the proportions could be influenced by non-tumor cells or infiltrating leukocytes that might express lower levels than tumor cells and in future studies it would be interesting to know what proportion of the cells were tumor cells and that this was similar in each sample.

In addition to assessing levels of the IR isoforms *in vivo*, we also looked *in vitro* and observed that three (LNCaP, PC-3, and VCaP) of our four cell lines displayed a similar profile to the cancer tissue and expressed more IR-A than IR-B. Only one PCa cell line we examined expressed more IR-B than A (DU145 cells). We then exposed PCa cells to different levels of glucose to mimic normal and hyperglycemic conditions: we observed that when DU145 cells were exposed to normal, physiologic levels of glucose (5 mM) they predominantly expressed the IR-A isoform but when exposed to hyperglycemic levels of glucose (25 mM) the IR-B isoform was the main isoform expressed. In the other PCa cell lines that we examined (LNCaP, VCaP, or PC3), all of which inherently contained less than 10% of their IR in the IR-B isoform, exposure to high glucose did not increase the low proportion of IR-B. We did, however, observe that the isoforms responded similarly to high glucose with an increase in IR-B in breast epithelial cells (MCF-10A and HS578T cells). So it appeared that for the majority of PCa cell lines the IR-A isoform predominated in either concentration of glucose, but in DU145 cells (and other non-prostate cell lines), the isoform ratio could be manipulated by increasing the concentrations of the glucose and switching from an IR-A to an IR-B dominant phenotype. An IR-B-dominant phenotype would presumably be more responsive to insulin and has been associated with a more differentiated phenotype in a number of different tissues, including adipocytes, hepatocytes, and in thyroid cancer (19–21). Furthermore, we did observe that a proportion of our paired patient samples also exhibited more IR-B than A in the cancer compared to the benign region. The IR-A isoform appears to be dominant in most cancer cell lines and *in vivo* which is also consistent with a reduction in the IR-B being associated with poorly differentiated cells, as observed in many cancers.

As insulin and IGF-II are the ligands for IR and their levels and/or availability may be increased in men with PCa who are obese or have type 2 diabetes: we also assessed alterations in IR isoforms following exposure to these agents in both concentrations of glucose in DU145 cells. Regardless of glucose concentrations, the addition of either IGF-II or insulin both reduced IR-B culminating in a relative increase in IR-A (that has a high affinity for IGF-II). In the PCa cell lines that expressed very low levels of IR-B, we observed that these levels did not respond to siRNA or change with altered glucose levels; this may be due to problems with the splicing mechanisms or could be due to instability of IR-B mRNA resulting in no observed changes in these cells. In the DU145 cell line, in which the IR-B isoform did increase in conditions of high glucose, upon exposure to IGF-II or insulin again the predominant isoform was IR-A. Expression of this isoform could potentially enable cancer cells to respond more effectively to IGF-II, which is known to be over-expressed by most cancers, including that of the prostate irrespective of glucose conditions. This may be a contributing factor to why obese patients that develop PCa have more aggressive disease and a higher mortality rate (22–24).

We also assessed the consequences of changes in IR isoform by examining alterations in phenotype in the DU145 cells in which the isoform ratio was altered following exposure to increased levels of glucose or suppressed by siRNA. We observed that insulin induced significantly more migration when the cells were exposed to high concentrations of glucose (higher IR-B) than it did in normal glucose conditions (higher IR-A) and conversely IGF-II promoted greater migration under the normal glucose conditions. While insulin was clearly more effective than IGF-II in hyperglycemic conditions (with higher IR-B), both were equally effective under normal glucose conditions. With proliferation, IGF-II again promoted a greater proliferative response in normal (high IR-A) compared to high glucose conditions (high IR-B). In conditions of high glucose, we observed a smaller response to insulin compared with IGF-II even though the IR-B isoform was predominant. However, it did induce a significant increase in proliferation under normal glucose conditions. IGF-II and insulin were both less effective in high compared to low glucose conditions; thus, hyperglycemia induced both relative insulin- and IGF-II-resistance in terms of proliferation. Clearly, exposure to different concentrations of glucose will affect many other aspects of cell function in addition to altering the IR isoforms.

To investigate the responses to insulin and IGF-II further, we successfully developed siRNA to effectively silence the IR-B isoform; which in high glucose conditions left IR-A as the predominant isoform. We initially observed that silencing IR-B alone significantly inhibited cell growth, suggesting a role for the IR-B isoform in cell proliferation. This was not observed by Heidegger et al. when they silenced IR-B but they performed their experiments in GM as opposed to SFM, which may account for the observed differences (25). Upon silencing IR-B (increasing relative abundance of the IR-A isoform) the cells showed a similar significant proliferative response to IGF-II but were no longer able to respond to insulin stimulation, indicating the reliance of insulin on the IR-B isoform for inducing an increase in cell growth. As with the exposure to different glucose concentrations, we were unable to silence the IR-B isoform in the other PCa cell lines (LNCaP, VCaP, or PC3) that already expressed predominantly IR-A, but were able to knockdown the IR-B isoform similarly in MCF-7 breast cancer cells. The abundance of the IR-B in these cell lines (LNCaP, VCaP, or PC3) is already very low (less than 10% of the IR-B isoform relative to total levels if the IR) and so it would be technically difficult to assess effective knockdown of IR-B by agarose gel electrophoresis. Interestingly, the MCF-7 breast cancer cell line, in which IR-B silencing was also effective has comparable abundance of IR-A and -B, with IR-B being the predominant isoform in hyperglycemic conditions. We also assessed the acute signaling response to IGF-II and insulin in the presence or absence of siRNA to IR-B. With Akt activation, we found that IR-B knockdown alone had no effect. IGF-II and insulin each induced a significant increase in Akt activation that was unaffected when IR-B was silenced. With IGF-II this response correlated with the cell counting data, where the proliferative response to IGF-II was also unaffected when IR-B was knocked down. However, while the ability of insulin to induce proliferation was blocked when IR-B was silenced the activation of Akt was not affected, suggesting that Akt activation at this time point was not mediating the proliferative response to insulin via IR-B. There were no significant effects of either IGF-II or insulin in the presence or absence of siRNA to IR-B on p-MAPK, suggesting an alternative pathway may be responsible for mediating the proliferative effects of insulin via IR-B in these cells following 30 min of treatment. The exposure time may be important in determining which pathways are activated as it has been reported that the internalization dynamics of IR-A and B (26) and the kinetics of signaling of the IGF-IR and IR (27) are different. Although insulin acutely activated Akt in the absence of IR-B to translate into a proliferative signal, it may need a more sustained signal. We have demonstrated that alternative splicing of the IR can be manipulated by different exposures and that the relative levels of these isoforms will influence how the cancer cells respond to both insulin and IGF-II. Alterations in the isoforms in response to changes in their hormonal milieu (as a result of the co-morbidities linked to a Western lifestyle) could have a profound impact on how malignant cells behave and play a role in promoting carcinogenesis. A greater understanding of the mechanisms underlying changes in alternative splicing of the IR may provide additional targets for future cancer therapies.

## Author Contributions

JW, HZ, AF, and CJ performed the research experiments. CP wrote the manuscript, contributed to the design, and supervised the study. JH contributed to the design, supervision, and to the writing of the paper. ML contributed in the supervision of the study. ML, AR, and JO accessed the tissue samples and supervised this aspect of the work. AB also contributed to writing the paper and provided invaluable advice regarding the potential clinical applications of the work.

## Conflict of Interest Statement

The authors declare that there is no conflict of interest that could be perceived as prejudicing the impartiality of the research reported.

## References

[1] Ebina Y Ellis L Jarnagin K Edery M Graf L Clauser E The human insulin receptor cDNA: the structural basis for hormone-activated transmembrane signalling. Cell (1985) 40:747–58. 10.1016/0092-8674(85)90334-4 2859121

[2] Ullrich A Bell JR Chen EY Herrera R Petruzzelli LM Dull TJ Human insulin receptor and its relationship to the tyrosine kinase family of oncogenes. Nature (1985) 313:756–61. 10.1038/313756a0 2983222

[3] Mosthaf L Grako K Dull TJ Coussens L Ullrich A McClain DA. Functionally distinct insulin receptors generated by tissue-specific alternative splicing. EMBO J (1990) 9:2409–13. 2369896 10.1002/j.1460-2075.1990.tb07416.x PMC552265

[4] Yamaguchi Y Flier JS Benecke H Ransil BJ Moller DE. Ligand-binding properties of the two isoforms of the human insulin receptor. Endocrinology (1993) 132:1132–8. 10.1210/en.132.3.1132 8440175

[5] Yamaguchi Y Flier JS Yokota A Benecke H Backer JM Moller DE. Functional properties of two naturally occurring isoforms of the human insulin receptor in Chinese hamster ovary cells. Endocrinology (1991) 129:2058–66. 10.1210/endo-129-4-2058 1655392

[6] Frasca F Pandini G Scalia P Sciacca L Mineo R Costantino A Insulin receptor isoform A, a newly recognized, high-affinity insulin-like growth factor II receptor in fetal and cancer cells. Mol Cell Biol (1999) 19:3278–88. 10.1128/MCB.19.5.3278 10207053 PMC84122

[7] Biernacka KM Perks CM Holly JM Role of the IGF axis in prostate cancer. Minerva Endocrinol (2012) 37:173–85. 22691890

[8] Chi KN Bjartell A Dearnaley D Saad F Schroder FH Sternberg C Castration-resistant prostate cancer: from new pathophysiology to new treatment targets. Eur Urol (2009) 56:594–605. 10.1016/j.eururo.2009.06.027 19560857

[9] Novosyadlyy R Leroith D. Insulin-like growth factors and insulin: at the crossroad between tumor development and longevity. J Gerontol A Biol Sci Med Sci (2012) 67:640–51. 10.1093/gerona/gls065 22421704

[10] Cox ME Gleave ME Zakikhani M Bell RH Piura E Vickers E Insulin receptor expression by human prostate cancers. Prostate (2009) 69:33–40. 10.1002/pros.20852 18785179

[11] Heni M Hennenlotter J Scharpf M Lutz SZ Schwentner C Todenhofer T Insulin receptor isoforms A and B as well as insulin receptor substrates-1 and -2 are differentially expressed in prostate cancer. PLoS One (2012) 7:e50953. 10.1371/journal.pone.0050953 23251408 PMC3519512

[12] Zeng L Biernacka KM Holly JM Jarrett C Morrison AA Morgan A Hyperglycaemia confers resistance to chemotherapy on breast cancer cells: the role of fatty acid synthase. Endocr Relat Cancer (2010) 17:539–51. 10.1677/ERC-09-0221 20356977

[13] Chen Z Lu W Garcia-Prieto C Huang P. The Warburg effect and its cancer therapeutic implications. J Bioenerg Biomembr (2007) 39:267–74. 10.1007/s10863-007-9086-x 17551814

[14] Hammarsten J Hogstedt B. Clinical, haemodynamic, anthropometric, metabolic and insulin profile of men with high-stage and high-grade clinical prostate cancer. Blood Press (2004) 13:47–55. 10.1080/08037050310025735 15083641

[15] Hammarsten J Hogstedt B. Hyperinsulinaemia: a prospective risk factor for lethal clinical prostate cancer. Eur J Cancer (2005) 41:2887–95. 10.1016/j.ejca.2005.09.003 16243513

[16] Bostwick DG High-grade prostatic intraepithelial neoplasia – the most likely precursor of prostate-cancer. Cancer (1995) 75:1823–36. 10.1002/1097-0142(19950401)75:7+<1823::AID-CNCR2820751612>3.0.CO;2-7

[17] Fu VX Dobosy JR Desotelle JA Almassi N Ewald JA Srinivasan R Aging and cancer-related loss of insulin-like growth factor 2 imprinting in the mouse and human prostate. Cancer Res (2008) 68:6797–802. 10.1158/0008-5472.CAN-08-1714 18701505 PMC4237281

[18] Bhusari S Yang B Kueck J Huang W Jarrard DF. Insulin-like growth factor-2 (IGF2) loss of imprinting marks a field defect within human prostates containing cancer. Prostate (2011) 71:1621–30. 10.1002/pros.21379 21432864 PMC3825178

[19] Entingh AJ Taniguchi CM Kahn CR. Bi-directional regulation of brown fat adipogenesis by the insulin receptor. J Biol Chem (2003) 278:33377–83. 10.1074/jbc.M303056200 12807888

[20] Kosaki A Webster NJ. Effect of dexamethasone on the alternative splicing of the insulin receptor mRNA and insulin action in HepG2 hepatoma cells. J Biol Chem (1993) 268:21990–6. 8408055

[21] Vella V Pandini G Sciacca L Mineo R Vigneri R Pezzino V A novel autocrine loop involving IGF-II and the insulin receptor isoform-A stimulates growth of thyroid cancer. J Clin Endocrinol Metab (2002) 87:245–54. 10.1210/jcem.87.1.8142 11788654

[22] Efstathiou JA Bae K Shipley WU Hanks GE Pilepich MV Sandler HM Obesity and mortality in men with locally advanced prostate cancer: analysis of RTOG 85-31. Cancer (2007) 110:2691–9. 10.1002/cncr.23093 17999404 PMC3047389

[23] Freedland SJ Obesity and prostate cancer: a growing problem. Clin Cancer Res (2005) 11:6763–6. 10.1158/1078-0432.CCR-05-1305 16203761

[24] Kristal AR Gong Z. Obesity and prostate cancer mortality. Future Oncol (2007) 3:557–67. 10.2217/14796694.3.5.557 17927521

[25] Heidegger I Ofer P Doppler W Rotter V Klocker H Massoner P. Diverse functions of IGF/insulin signaling in malignant and noncancerous prostate cells: proliferation in cancer cells and differentiation in noncancerous cells. Endocrinology (2012) 153:4633–43. 10.1210/en.2012-1348 22903612

[26] Giudice J Leskow FC Arndt-Jovin DJ Jovin TM Jares-Erijman EA. Differential endocytosis and signaling dynamics of insulin receptor variants IR-A and IR-B. J Cell Sci (2011) 124:801–11. 10.1242/jcs.076869 21303927

[27] Foti M Moukil MA Dudognon P Carpentier JL. Insulin and IGF-1 receptor trafficking and signalling. Novartis Found Symp (2004) 262:125–41. 10.1002/0470869976.ch8 15562826

