# Supplementary material for: Insulin Receptor Isoform Variations in Prostate Cancer Cells
Source: Front Endocrinol (Lausanne). 2016 Sep 28;7:132. doi: 10.3389/fendo.2016.00132 (PMC5039983; doi:10.3389/fendo.2016.00132)
Supplement: Supplementary file 1 [file Image_1.PDF]

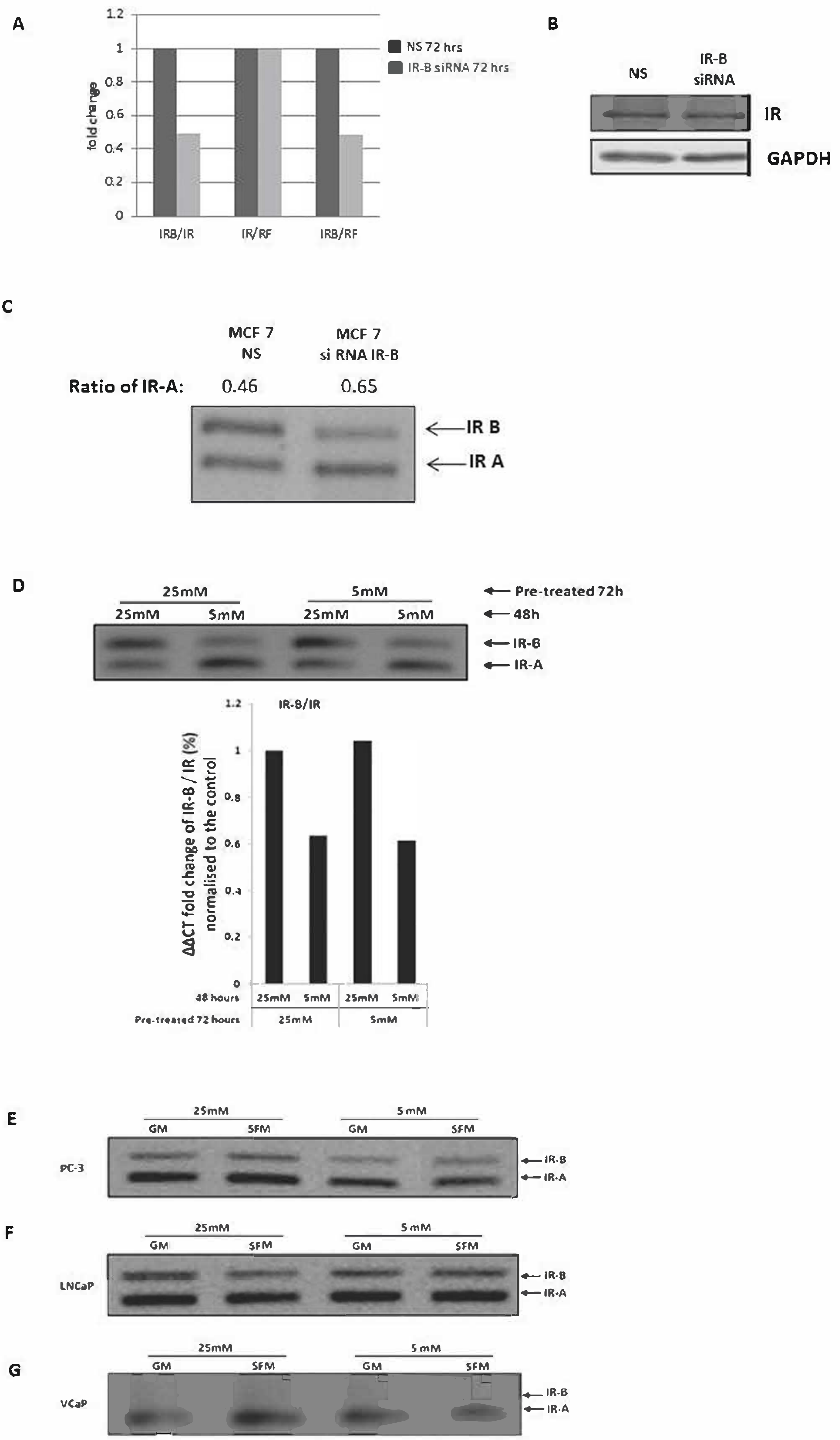

Supplementary Figure Legend 1 - (A&B) Representative q-PCR and western blot respectively to show IR abundance following siRNA silencing (50nM) of the IR-B isoform: for q-PCR IR-B was assessed relative to the IR and reference gene and IR was assessed relative to the reference gene. GAPDH was used as a loading control for the western blot. These are representative experiments. **(C)** MCF-7 cells were cultured in T25 flasks with 25mM GM at a density 6 ×105 per flask in the presence of IR-B siRNA. Non-silenced siRNA (NS) was used as a negative controls. After 72 hours the isoforms were assessed by RT-PCR (n=2). A representative blot is shown. **(D)** DU145 cells were seeded at a density of 0.3×106 cells per T25 flask in 5mM glucose GM for 24 hours before being exposed to either 25mM or 5mM glucose-containing SFM. After 72 hours pre-treatment all 25mM SFM flasks were changed into either 25mM and 5mM and all 5mM flasks were changed into either 5 or 25 mM glucose-containing SFM for another 48 hours. RNA was extracted and reversed into cDNA. RT-PCR and Q-PCR (D) were used to assess changes in the IR isoform ratio (n=1). **(E, F & G)** The effect of hyperglycaemia on IR isoforms was also investigated in PC-3, LNCaP and VCaP cells respectively. The IR-A: IR-B ratio was assessed by RT-PCR after 48 hours in different serum and glucose conditions. These are representative experiments.
